# Dysbiosis is not present in horses with fecal water syndrome when compared to controls in spring and autumn

**DOI:** 10.1111/jvim.15778

**Published:** 2020-06-26

**Authors:** Angelika Schoster, J. Scott Weese, Vinzenz Gerber, Claudia Nicole Graubner

**Affiliations:** ^1^ University of Zurich Equine Department Zurich Switzerland; ^2^ University of Guelph, University of Guelph Guelph Ontario Canada; ^3^ Vetsuisse Faculty, University of Bern Department of Veterinary Medicine Bern Switzerland; ^4^ Equine Clinic ‐ Vetsuisse Faculty, University of Bern Department of Veterinary Medicine Berne Switzerland

**Keywords:** clostridiales, fecal water syndrome, gastrointestinal microbiota, horse, metagenomic sequencing

## Abstract

**Background:**

Fecal water syndrome (FWS) is long‐standing and common in horses, particularly in central Europe. No large epidemiological data sets exist, and the cause remains elusive. Dysbiosis could play a role in pathogenesis.

**Objectives:**

To evaluate whether dysbiosis is present in horses with FWS when compared to stable‐matched control horses in spring and autumn.

**Animals:**

Fecal samples were collected from horses with FWS (n = 16; 9 mares, 7 geldings) and controls (n = 15; 8 mares, 7 geldings).

**Methods:**

The bacterial microbiome of samples collected in spring and autumn of 2016 was analyzed using high‐throughput sequencing. Differences in relative abundance of bacterial taxa, alpha diversity, and beta diversity indices were assessed between horses with FWS and controls based on season.

**Results:**

Differences in microbial community composition based on time point and health status were not observed on any taxonomic level. Limited differences were seen on linear discriminant analysis effect size analysis. No difference in alpha diversity indices was observed including richness, diversity based on health status, or time point. No effect of health status on microbial community membership structure was observed.

**Conclusions and Clinical Importance:**

Limited differences were found in the bacterial microbiota of horses with and without FWS, regardless of season. Further research is needed to elucidate the role of microbiota in the development of FWS.

**Abbreviations**
AMOVA: analysis of molecular variance
ANOSIM: analysis of similarities
bp: base pairs
EGUS: equine gastric ulcer syndrome
FDR: false discovery rate
LEfSe: linear discriminant analysis effect size
OTU: operational taxonomic unit
PCoA: principal coordinate analysis
RDP: ribosomal database project

## 1 INTRODUCTION

Fecal water syndrome (FWS) in horses is a common problem, but so far it is reported in the literature only in central Europe.^1^ Horses with FWS defecate manure of normal consistency, but watery fecal content also is passed before, during, or after defecation.^2^ Horses usually do not show signs of severe intestinal disease, but some show mild signs of discomfort during defecation. In severe cases, weight loss, dermatitis along the hind limbs, and a general decline in condition also can be observed.^1^ No large epidemiological data sets exist, but FWS has been reported to occur in adult horses of all breeds and signs occur most commonly in winter. The cause remains elusive, and organic disease in affected horses usually cannot be found. No association has been found with dentation status, feeding management, and parasite load.^2^ Other suggested causes include dysbiosis, inflammation, and stress.^2^ The owners of affected horses report that FWS can be severe enough to disable the horses. Veterinary examinations frequently fail to identify any underlying pathology.

The bacterial microbiota plays a key role in human and animal health. Recent studies indicate that imbalances in the microbial communities and their function (dysbiosis) can be associated with diseases in the gastrointestinal tract and beyond.^3^ Dysbiosis in horses has been associated with colitis,^4^ laminitis,^5^, ^6^ grass sickness,^7^ colic,^8^ and diarrhea in foals.^9^, ^10^ Although dysbiosis has been suggested as a contributing factor in FWS in horses, this association has not been studied.

Our objective was to analyze differences in the fecal bacterial microbiota between horses with FWS and stable‐matched healthy controls at 2 time points.

## 2 METHODS

This study was conducted with the approval of the Swiss Cantonal Veterinary Office (BE100/13).

### 2.1 Animals and study protocol

A prospective case‐control study was performed. Referring veterinarians of the equine clinic of the University of Bern were asked to provide contact information of owners of horses suffering from FWS. Horse owners then were called by a veterinary student in her final year (LC) and asked to take part in a telephone survey. The questionnaire contained in‐depth information about the horses' health status in general, use, and the individual history of the FWS (Supplementary Information S4). Horses were included in the study if the FWS duration was >4 months, fecal water was passed at least on 1 of 2 days during a period of at least 1 week, and if a physical examination performed by 2 veterinary students in their final year (LC, NB) was normal. A matched control was selected for every case but in 2 stables 2 patients were compared to 1 control, and in 1 stable 1 patient was compared to 2 controls. The inclusion criterion for control horses was absence of FWS. The control horses were the same age and same breed as the horses with FWS and had to have direct contact with the FWS horses or be stall neighbors to ensure consumption of the same water.

### 2.2 Sample collection

Fecal samples were collected during early spring by 1 of the authors (LC, February‐March 2014) and in autumn (August‐September 2014) of the same year. The autumn samples were collected by the owners after providing a fecal container at the spring visit. The packages were shipped overnight to the laboratory. The time points were chosen because FWS in this population was more frequent during spring than during autumn based on results of the telephone questionnaires. The samples were stored at 4°C for a maximum of 2 weeks before being transported to the laboratory where all were frozen at −80°C until DNA extraction. Some horses also had gastroscopy, measurement of saliva cortisol concentration after ACTH stimulation,^11^ and fecal testing based on McMaster technique performed (Supplemental Information).

### 2.3 Metagenomic analysis of fecal samples

A sample of DNA was extracted from 1 g feces using a commercial kit (E.Z.N.A. Stool DNA Kit, Omega Bio‐Tek Inc, Georgia) according to manufacturer's recommendations. Adequate DNA quality and quantity were assessed by spectrophotometry (NanoDrop, Roche, Ontario, Canada).

Amplification of the V4 region of the 16S rRNA gene, purification, and sequencing were performed as previously described.^12^ Briefly, primers targeting the V4 region of the 16S rRNA gene were designed with overhanging adapters for annealing to the Illumina index primers in the second PCR step. The PCR products were purified and Illumina index primers were attached during the second PCR step. The PCR products were purified and evaluated by gel‐electrophoresis in 1.5% agarose gel. The samples were sequenced at the University of Guelph's Advanced Analysis Centre using an Illumina MiSeq (Illumina RTA v1.17.28; MCS v2.2).

### 2.4 Bioinformatics and statistics

Distribution of age, sex, breed, equine gastric ulcer syndrome (EGUS) grade, fecal egg counts, and salivary cortisol concentrations were compared between horses with and without FWS using Fisher's exact or chi square tests.

Sequence processing was performed using Mothur 1.42.3 as outlined in the Mothur MiSeq SOP (https://www.mothur.org/wiki/MiSeq_SOP).^13^ Primers were removed and sequences were aligned into contigs using the default Mothur settings. Contigs underwent a series of quality control steps, including removal of sequences with ambiguous base calls that were of inappropriate length (<239 or >244 base pairs) or contained runs of homopolymers >8 base pairs. Contigs were aligned to the SILVA 16S rRNA reference data^14^ to ensure sequences were consistent with the V4 region. Those that did not properly align were removed. Chimeras were detected using UCHIME^15^ and removed. Contigs were identified using ribosomal database project (RDP) classifier.^16^ Contigs identified as chloroplast, mitochondria, archaea, or eukaryote were removed (0.3% of readings). Contigs were binned into operational taxon units (OTUs) using an open (de novo) OTU picking approach at a 3% dissimilarity level.^17^ The OTUs also were identified using the RDP classifier (http://rdp.cme.msu.edu/index.jsp). Subsampling to the level of the sample with the smallest number of sequences (ie, 134 584) was performed to normalize sequence numbers for further comparison. Subsampling therefore consisted of random selection of 134 583 sequences from each sample. Completeness of sampling effort was assessed using Good's coverage. The sequences were uploaded to the University's data verse server (https://doi.org/10.5683/SP2/A5NZZP).

Alpha diversity was described using Chao richness, Shannon's evenness, and inverse Simpson's index. Only bacterial taxa accounting for >1% of the total were used for statistical analysis. Data were determined to be nonparametric based on examination of quantile plots and Shapiro‐Wilk testing. Relative abundances and alpha diversity indices were compared between healthy horses and horses with fecal water and between time points using the Wilcoxon test. False discovery rate (FDR) adjustments were performed using the Benjamin Hochberg procedure for comparisons of relative abundance of taxa.

Community overlap and structure were compared between groups by parsimony test, analysis of molecular variance (AMOVA), and analysis of similarities (ANOSIM) applied to the Jaccard (community membership) and Yue and Clayton (community structure) indices, respectively. Dissimilarity was visualized using principal coordinate analysis (PCoA). Linear discriminant analysis (LDA) effect size (LEfSe) analysis, involving Wilcoxon and Kruskal‐Wallis tests was performed to identify differentially abundant OTUs with 97% sequence similarity between groups.^18^ The LDA value threshold was 2.0. A *P*‐value of <.05 was considered significant for all comparisons. A commercial program was used for all statistical analyses (JMP Statistical discoveries, Version 11).

## 3 RESULTS

### 3.1 Demographic data of animals

Thirty‐one horses were included, 16 horses with FWS and 15 stable‐matched controls. Age, sex, breed, and farm distribution are presented in Supplemental Information. Nine of 16 affected horses were female (56%) and middle‐aged (median, 14.2 years; range, 3‐32 years). No statistical difference was found among age, breed, and sex between control horses and horses with fecal water (*P* = .16, .57, and .15, respectively). The main breeds represented in the FWS group in our study were breeds used for pleasure riding including Freiberger, Frisian, and Frisian mix breeds, Fjord ponies, PRE and Haflinger (Supplementary Information S1). The owners of 8/16 (50%) of horses with FWS reported that fecal water occurred during winter or spring, whereas 3/16 (19%) reported that fecal water occurred during the whole year. The others reported no specific season but intermittent fecal water. Results from gastroscopy, ACTH stimulation testing and saliva cortisol measurements, and McMaster fecal analysis are presented in Supplementary Information S1. No significant difference was found between affected horses and controls with regard to EGUS grade (*P* = .6) and McMaster egg count (*P* = .8). Salivary cortisol concentration after ACTH stimulation was normal in all tested horses and therefore comparison could not be performed.

### 3.2 Sequencing quality data

A total of 4 097 088 V4 16S RNA gene sequences passed all quality control filters. Sequence numbers ranged from 134 584 to 552 218 (median 45 944). Median sequence numbers were 44 300 (range, 17 775‐5 542 178) in the control group and 48 067 (range 134 584‐375 450) in the FWS group.

### 3.3 Microbial community composition

**FIGURE 1:**
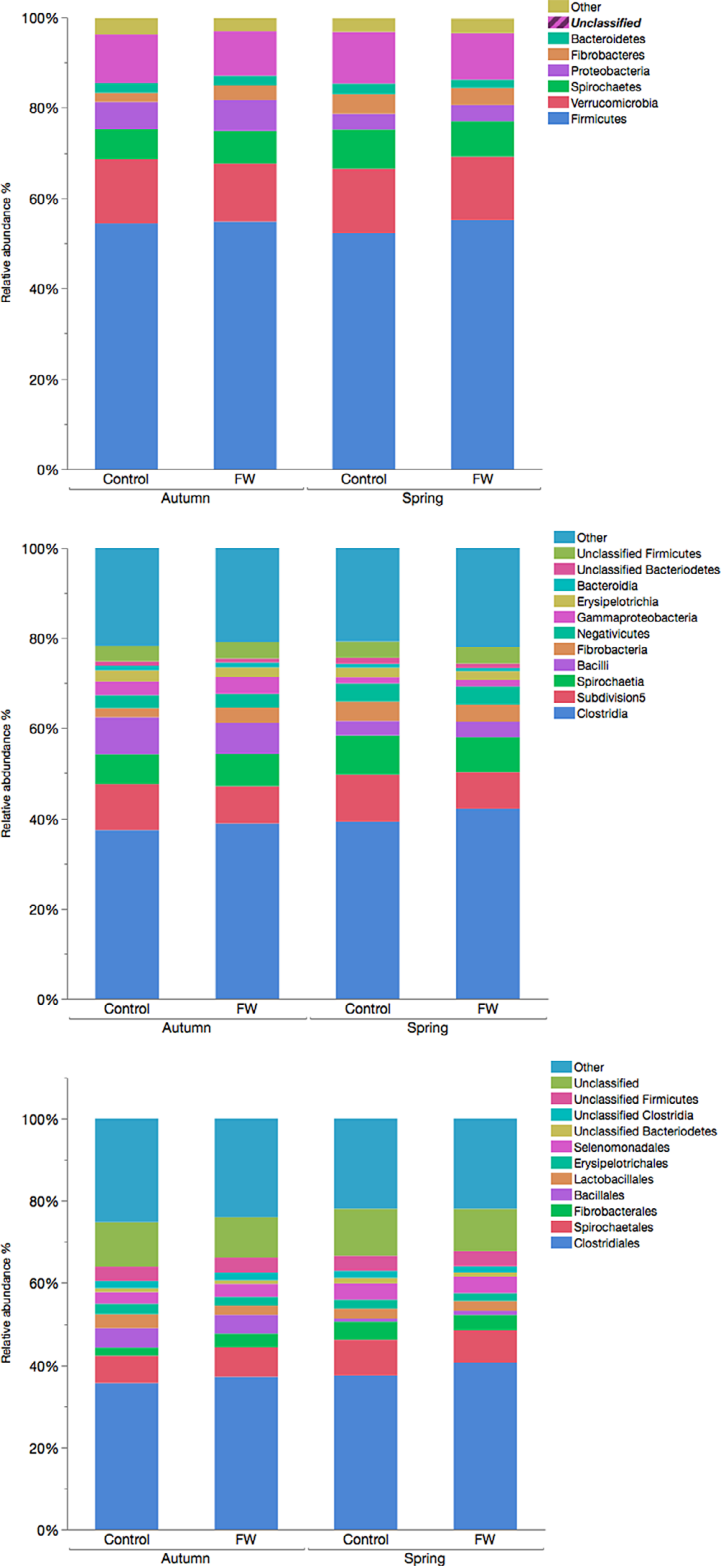
Relative abundance of A, phyla, B, classes, and C, orders of horses with and without fecal water syndrome sampled in autumn and spring. Other includes taxa with a relative abundance of <1%. In spring, 15 and 16 horses with and without fecal water were sampled, respectively; in autumn, 9 and 7 horses with and without fecal syndrome were sampled, respectively

Thirty different phyla, 80 classes, and 137 orders were identified. Seven phyla, 11 classes, and 12 orders had a mean relative abundance >1% (Figure 1).

### 3.4 Fecal microbial composition compared between horses with FWS and matched controls

Analysis was stratified based on time point of sample collection. Only taxa with a relative abundance >1% were analyzed. No significant differences were found between the groups on the phylum, class, or order level after FDR adjustment (all *P* > .3, Figure 1).

### 3.5 Linear discriminant analysis effect size

**FIGURE 2:**
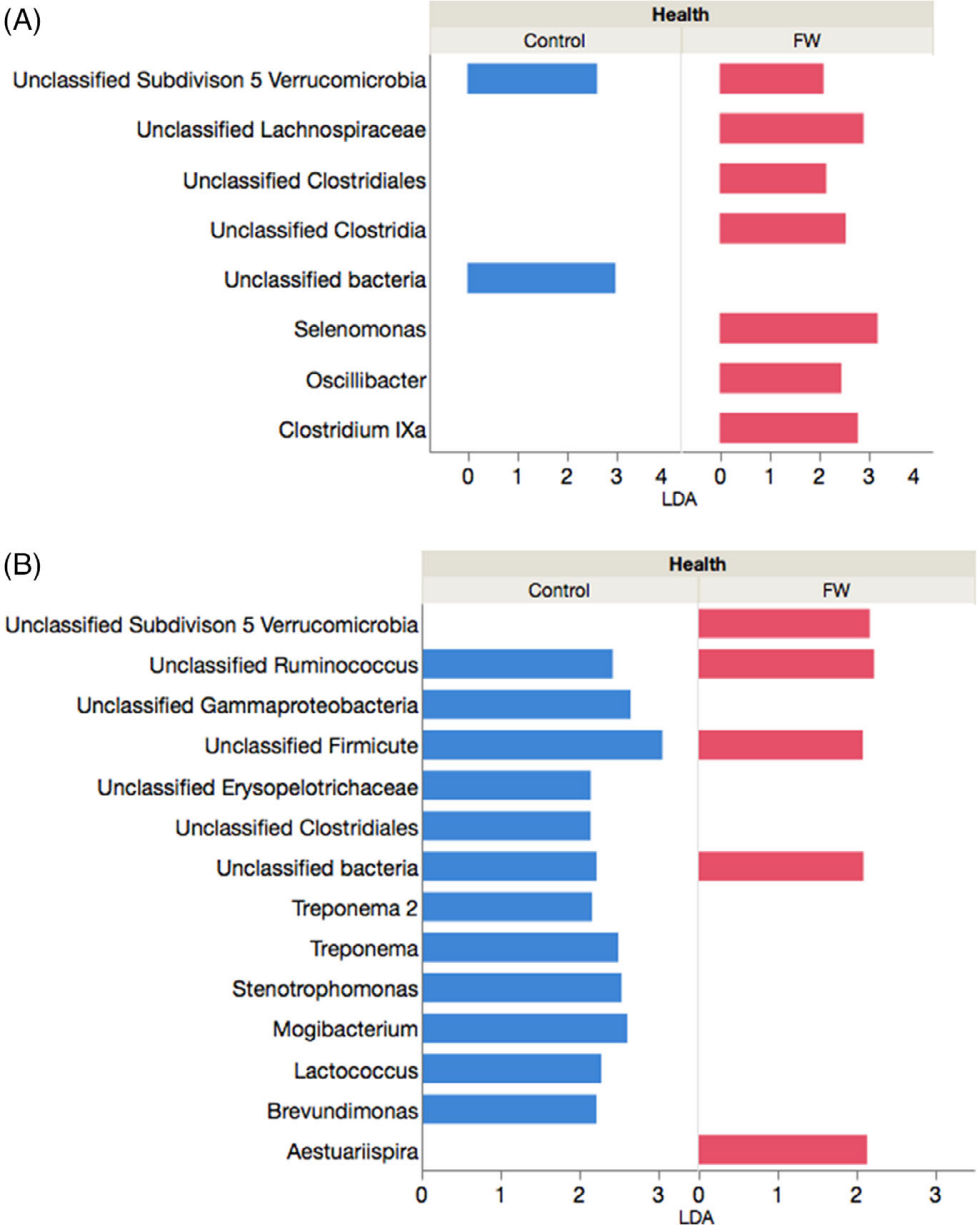
Species significantly enriched in the bacterial microbiota of horses with (red) and without (blue) fecal water syndrome sampled in A, spring and B, autumn determined by linear discriminant analysis effect size (LEfSe). In spring, 15 and 16 horses with and without fecal water were sampled, respectively; in autumn, 9 and 7 horses with and without fecal syndrome were sampled, respectively

In spring, 2 taxa were identified as enriched using LEfSe in the healthy group whereas 7 taxa were enriched in the FWS group (Figure 2A, full list, *P* values, and LDA values are shown in Supplementary Information S2). In autumn, 12 taxa were enriched in the control group whereas 5 taxa were enriched in the FWS group (Figure 2B, full list, *P* values, and LDA values are shown in Supplementary Information S3). Several enriched species also occurred in fall and spring when the population as a whole was examined, and when each subgroup (FWS and controls) was analyzed on its own (Supplementary Information S3).

### 3.6 Alpha diversity

No differences were found in alpha diversity indices between horses with FWS and control horses and both time points (Table 1).

**TABLE 1:** Alpha diversity indices and estimate of coverage of the microbiota of horses with fecal water syndrome and stable‐matched controls. In spring, 16 and 15 horses with and without fecal water were sampled, respectively; in autumn, 8 and 7 horses with and without fecal syndrome were sampled, respectively

	Time point	Median (range)		*P*‐value
		Control	Fecal water	
Chao richness index	Spring	7576.2 (5740.6‐15 991.3)	9952.7 (4982.7‐18 977.8)	.58
	Autumn	10 614.8 (6537.6‐18 735.1)	7084.6 (4689.2‐12 362.2)	.07
Simpson diversity index	Spring	375.5 (144.1‐908.1)	400.2 (172.2‐685.9)	.67
	Autumn	464.1 (133.1‐582.6)	311.1 (81.7‐744.5)	.36
Shannon evenness index	Spring	0.88 (0.84‐0.91)	0.88 (0.85‐0.91)	.85
	Autumn	0.89 (0.82‐0.89)	0.86 (0.79‐0.91)	.31
Good's coverage	Spring	0.82 (0.67‐0.85)	0.79 (0.66‐0.87)	.58
	Autumn	0.78 (0.68‐0.84)	0.83 (0.73‐0.88)	.11

### 3.7 Beta diversity

**FIGURE 3:**
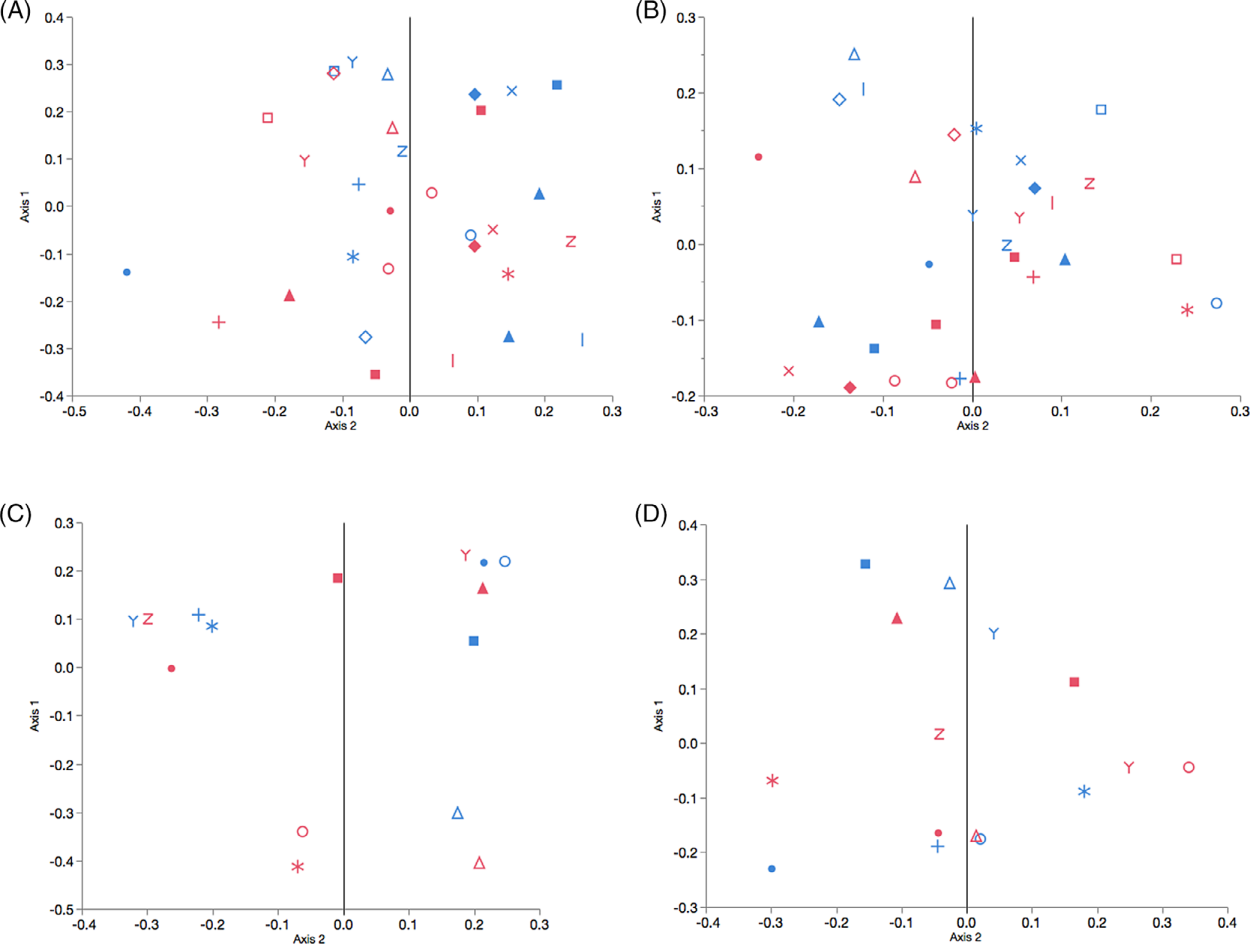
Principal coordinate analysis of the fecal bacterial microbiota of horses with and without fecal water syndrome sampled in spring (A, B) and autumn (C, D). Principal coordinate analysis based on the Jaccard index in spring (B) and autumn (D) and the Yue and Clayton index in spring (A) and autumn (C); red: fecal water syndrome, blue: control. Each symbol represents a farm (disease/control pairs). In spring, 16 and 15 horses with and without fecal water were sampled, respectively; in autumn, 8 and 7 horses with and without fecal syndrome were sampled, respectively. Each symbol represents a farm, in 2 farms 2 horses were compared to 1 control and in 1 farm 1 horse was compared to 2 controls

Distinct clusters (FWS versus controls) were not visually evident on Jaccard and Yue and Clayton PCoA (Figure 3) at either time points, and a statistically significant difference in community structure was not seen across statistical tests (Table 2).

**TABLE 2:** Difference in bacterial microbial composition (Jaccard index) and structure (Yue and Clayton index) of the fecal bacterial microbiota of horses with fecal water syndrome and stable‐matched controls. In spring, 16 and 15 horses with and without fecal water were sampled, respectively; in autumn, 8 and 7 horses with and without fecal syndrome were sampled, respectively

	Spring		Autumn	
	Jaccard index *P*‐value	Yue and Clayton index *P*‐value	Jaccard index *P*‐value	Yue and Clayton index *P*‐value
Parsimony	.92	.19	.89	.57
UniFrac	.99	.96	.99	.96
AMOVA	.82	.60	.88	.61
ANOSIM	.64	.79	.91	.96

Abbreviations: AMOVA, analysis of molecular variance; ANOSIM, analysis of similarities.

## 4 DISCUSSION

Fecal water syndrome is common in horses in Europe. Dysbiosis has been suggested to play role in FWS in horses, but this possibility has not been studied to date. We found only minor differences in microbiota composition between horses with FWS and stable‐matched controls. Alpha and beta diversity did not differ between affected horses and stable‐matched controls.

### 4.1 Microbiota composition of horses affected with FWS

No difference was seen in alpha diversity between horses affected with FWS and stable‐matched controls. In humans with functional gastrointestinal disorders, particularly irritable bowel syndrome, the diversity of microbial populations is decreased.^19^, ^20^ A potential explanation for the lack of difference seen in our study could be the power of the study.

Fecal water syndrome likely is a multifactorial disease. Stress associated with low hierarchical status in the herd has been suggested because it is a factor for the development of FWS.^2^ A link between dysbiosis and stress has been suggested because animal models have shown that acute and chronic stress can alter intestinal permeability and induce inflammation in the gastrointestinal system.^21^, ^22^ Results from the ACTH stimulation stress test and gastroscopy could not confirm stress as a reason for FWS in this population.

The microbiota produces a substantical proportion of the gases present in the gastrointestinal tract, including hydrogen sulfide. Hydrogen sulfide recently has been recognized as a gaseous neuromodulator or neurotransmitter capable of modulating intestinal inflammation.^23^ The term microbiota‐gut‐brain axis has been coined to describe this important relationship among these 3 organs.^24^ Many bacteria can produce hydrogen sulfide, including some of the bacteria enriched in the FWS group in our study (*Selenomonas*).^25^ Additional research could focus on the role of metabolites produced by the microbiota in addition to the composition of the microbiota.

Differences based on health status and season were evident on LEfSe analysis. Statistical tests dependent on taxonomic categories such as phyla and order often fail to detect community‐level differences because they mainly take into account relative abundances.^26^ Approaches thus have been developed to evaluate community‐based measures for evaluating the microbiota, such as community membership, community structure, and other ways to assess differences in composition such as LEfSe. This method identifies features that are statistically different among biological classes using a nonparametric test and then applies additional tests to assess whether these differences are consistent with regard to expected biological behavior. The LEfSe analysis therefore emphasizes both statistical significance and biological relevance.^18^ Lachnospiraceae and Ruminococcaceae, members of the Clostridia class, are consistently underrepresented in studies of humans and animals, independent of the cause of gastrointestinal disease.^27^, ^28^, ^29^, ^30^, ^31^ A decrease of these bacterial families in horses with colitis and colic as well as horses exposed to stressful factors such as fasting, transport, and anesthesia also has been found,^4^, ^8^, ^32^ and a decrease in some of these members has been identified before the onset of colic in postpartum mares.^8^ Unfortunately >50% of the enriched species were unclassified in our study, limiting interpretation of the results. Several OTUs belonging to the Lachnospiraceae and Ruminococcaceae were enriched in the FWS group as well as in the control group. A decrease in these species that are important for gastrointestinal health in horses with FWS therefore was not supported in our study.

In healthy horses, temporal variation is limited and differences usually are based on individual composition of the microbiota and husbandry, particularly feeding practices.^33^, ^34^ We elected to sample horses and their controls at 2 time points to try and collect samples when signs were most severe and during more normal time periods. Sampling at more time points would be necessary to assess whether temporal stability is decreased in horses with FWS compared to healthy controls. One limitation was that approximately 50% of horses were unavailable for repeat sampling in autumn.

There is individual animal variation in the composition of the microbiota in horses, but there is also a substantial impact of husbandry. Different feeding and management conditions have been shown to cause significant shifts in the microbiota.^33^ We therefore chose to include stable, age, and breed‐matched controls to decrease the bias of husbandry.

## 5 LIMITATIONS

The main limitation of our study was the selection of cases and controls. Because different diseases have been suggested as causes for chronic diarrhea and also for individual FWS cases, a standardized approach to diagnose FWS does not exist. Therefore, we chose to include horses for which the veterinarian in charge of the case perceived the horse to be suffering from FWS and for which the history was consistent with this syndrome. Additional tests for the patients depended on the cost to the owners and therefore could not be regarded as inclusion criteria. Controls were selected based on normal manure production, normal clinical examination, and similar age and breed. The abnormal findings for some of the diagnostic tests (eg, presence of EGUS on gastroscopy) were not considered as the cause of FWS, but this possibility could not be ruled out completely.

Storage of fecal samples at 4°C for 14 days is not ideal because data from human medicine indicate stability for 24 hours at 4°C, and current recommendations are to store samples for a maximum of 24 hours at 4°C.^35^, ^36^ However, storage at 4°C for 14 days had a limited effect on microbial composition of feces in dogs and cats when assessed by metagenomic sequencing.^37^ No data are available for fecal samples from horses. Disease status also might play a role in the stability of the microbiome under different storage conditions. In a study comparing different sampling and storage methods in healthy human subjects and patients with inflammatory bowel disease, sampling method had an effect whereas storage method did not.^35^ No difference was found between the reference storage method (immediate storage at −80°C) compared to room temperature for 24 hours, 4°C for 24 hours, and −20°C for 2 weeks, with regard to changes in the relative abundance of dominant phyla, relative abundance of oxygen sensitive species, alpha diversity indices, and microbial structure. The effect of different storage methods was also not significant in the subgroups of healthy individuals and patients with inflammatory bowel disease. Our samples, however, were stored for 14 days at 4°C, and it is unclear how the prolonged storage at 4°C might have affected microbial composition.

Fecal water syndrome is likely a multifactorial disease. Minor changes in the gut microbiota were present between affected horses and healthy horses, indicating that the microbiota is a potential factor in the development of clinical signs. Additional research is needed to further elucidate the role of the microbiota in FWS and to try and exploit this knowledge for new therapeutic strategies.

## CONFLICT OF INTEREST DECLARATION

Authors declare no conflict of interest.

## OFF‐LABEL ANTIMICROBIAL DECLARATION

Authors declare no off‐label use of antimicrobials.

## INSTITUTIONAL ANIMAL CARE AND USE COMMITTEE (IACUC) OR OTHER APPROVAL DECLARATION

This study was conducted with the approval of the Swiss cantonal veterinary office (BE100/13).

## HUMAN ETHICS APPROVAL DECLARATION

Authors declare human ethics approval was not needed for this study.

## Supporting information


**Appendix** **S1.** Supplemental Table 1. Information to dysbiosis not present in horses with fecal water syndrome when compared to controls in spring and autumn: Population data on horses studied

**Appendix** **S2**. Supporting Information.

**Appendix** **S3**. Supplementary Table 2. LEFSE results

**Appendix** **S4**. Supporting Information.

## References

[1] Ertelt A , Gehlen H . Free fecal water in the horse ‐ an unsolved problem. Pferdeheilkunde. 2015;31:261‐268.

[2] Kienzle E , Zehnder C , Pfister K , Gerhards H , Sauter‐Louis C , Harris P . Field study on risk factors for free fecal water in pleasure horses. J Equine Vet Sci. 2016;44:32‐36.

[3] Costa MC , Weese JS . The equine intestinal microbiome. Animal health research reviews/Conference of Research Workers in Animal Diseases; 2012:1‐8. 10.1017/S1466252312000035 22626511

[4] Costa MC , Arroyo LG , Allen‐Vercoe E , et al. Comparison of the fecal microbiota of healthy horses and horses with colitis by high throughput sequencing of the V3‐V5 region of the 16S rRNA gene. PLoS One. 2012;7:e41484. 22859989 10.1371/journal.pone.0041484 PMC3409227

[5] Milinovich GJ , Trott DJ , Burrell PC , et al. Fluorescence in situ hybridization analysis of hindgut bacteria associated with the development of equine laminitis. Environ Microbiol. 2007;9:2090‐2100. 17635552 10.1111/j.1462-2920.2007.01327.x

[6] Steelman SM , Chowdhary BP , Dowd S , Suchodolski J , Janečka JE . Pyrosequencing of 16S rRNA genes in fecal samples reveals high diversity of hindgut microflora in horses and potential links to chronic laminitis. BMC Vet Res. 2012;8:231. 23186268 10.1186/1746-6148-8-231 PMC3538718

[7] Garrett LA , Brown R , Poxton IR . A comparative study of the intestinal microbiota of healthy horses and those suffering from equine grass sickness. Vet Microbiol. 2002;87:81‐88. 12079749 10.1016/s0378-1135(02)00018-4

[8] Weese JS , Holcombe SJ , Embertson RM , et al. Changes in the faecal microbiota of mares precede the development of postpartum colic. Equine Vet J. 2014;47:641‐649. 25257320 10.1111/evj.12361

[9] Kuhl J , Winterhoff N , Wulf M , et al. Changes in faecal bacteria and metabolic parameters in foals during the first six weeks of life. Vet Microbiol. 2011;151:321‐328. 21511405 10.1016/j.vetmic.2011.03.017

[10] Schoster A , Staempfli HR , Guardabassi LG , Jalali M , Weese JS . Comparison of the fecal bacterial microbiota of healthy and diarrheic foals at two and four weeks of life. BMC Vet Res. 2017;13:144. 28558788 10.1186/s12917-017-1064-x PMC5450145

[11] Scheidegger MD , Gerber V , Ramseyer A , Schüpbach‐Regula G , Bruckmaier RM , van der Kolk JH . Repeatability of the ACTH stimulation test as reflected by salivary cortisol response in healthy horses. Domest Anim Endocrin. 2016;57:43‐47. 10.1016/j.domaniend.2016.04.002 27565229

[12] Schoster A , Guardabassi L , Staempfli HR , et al. The longitudinal effect of a multi‐strain probiotic on the intestinal bacterial microbiota of neonatal foals. Equine Vet J. 2016;48(6):689–696. 10.1111/evj.12524. 26509834

[13] Schloss PD , Westcott SL , Ryabin T , et al. Introducing mothur: open‐source, platform‐independent, community‐supported software for describing and comparing microbial communities. Appl Environ Microbiol. 2009;75:7537‐7541. 19801464 10.1128/AEM.01541-09 PMC2786419

[14] Quast C , Pruesse E , Yilmaz P , et al. The SILVA ribosomal RNA gene database project: improved data processing and web‐based tools. Nucleic Acids Res. 2013;41:D590‐D596. 23193283 10.1093/nar/gks1219 PMC3531112

[15] Edgar RC , Haas BJ , Clemente JC , Quince C , Knight R . UCHIME improves sensitivity and speed of chimera detection. Bioinformatics. 2011;27:2194‐2200. 21700674 10.1093/bioinformatics/btr381 PMC3150044

[16] Wang Q , Garrity GM , Tiedje JM , Cole JR . Naive Bayesian classifier for rapid assignment of rRNA sequences into the new bacterial taxonomy. Appl Environ Microbiol. 2007;73:5261‐5267. 17586664 10.1128/AEM.00062-07 PMC1950982

[17] Morgan XC , Huttenhower C . Chapter 12: human microbiome analysis. PLoS Comput Biol. 2012;8:e1002808. 23300406 10.1371/journal.pcbi.1002808 PMC3531975

[18] Segata N , Izard J , Waldron L , et al. Metagenomic biomarker discovery and explanation. Genome Biol. 2011;12:R60. 21702898 10.1186/gb-2011-12-6-r60 PMC3218848

[19] Codling C , O'Mahony L , Shanahan F , Quigley EMM , Marchesi JR . A molecular analysis of fecal and mucosal bacterial communities in irritable bowel syndrome. Dig Dis Sci. 2010;55:392‐397. 19693670 10.1007/s10620-009-0934-x

[20] Carroll IM , Ringel‐Kulka T , Siddle JP , Ringel Y . Alterations in composition and diversity of the intestinal microbiota in patients with diarrhea‐predominant irritable bowel syndrome. Neurogastroenterol Motil. 2012;24:521‐530. 22339879 10.1111/j.1365-2982.2012.01891.x PMC3975596

[21] Saunders PR , Santos J , Hanssen NP , et al. Physical and psychological stress in rats enhances colonic epithelial permeability via peripheral CRH. Dig Dis Sci. 2002;47:208‐215. 11852879 10.1023/a:1013204612762

[22] Bradesi S , Schwetz I , Ennes HS , et al. Repeated exposure to water avoidance stress in rats: a new model for sustained visceral hyperalgesia. Am J Physiol Gastrointest Liver Physiol. 2005;289:G42‐G53. 15746211 10.1152/ajpgi.00500.2004

[23] Distrutti E , Sediari L , Mencarelli A , et al. Evidence that hydrogen sulfide exerts antinociceptive effects in the gastrointestinal tract by activating K‐ATP channels. J Pharmacol Exp Ther. 2006;316:325‐335. 16192316 10.1124/jpet.105.091595

[24] Cryan JF , O'Riordan KJ , Cowan CSM , et al. The microbiota‐gut‐brain axis. Physiol Rev. 2019;99:1877‐2013. 31460832 10.1152/physrev.00018.2018

[25] Linden DR . Hydrogen sulfide signaling in the gastrointestinal tract. Antioxid Redox Signal. 2014;20:818‐830. 23582008 10.1089/ars.2013.5312 PMC3910452

[26] MacLean D , Jones JD , Studholme DJ . Application of “next‐generation” sequencing technologies to microbial genetics. Nat Rev Microbiol. 2009;7:287‐296. 19287448 10.1038/nrmicro2122

[27] Suchodolski JS , Markel ME , Garcia‐Mazcorro JF , et al. The fecal microbiome in dogs with acute diarrhea and idiopathic inflammatory bowel disease. PLoS One. 2012;7:e51907. 23300577 10.1371/journal.pone.0051907 PMC3530590

[28] Honneffer JB , Minamoto Y , Suchodolski JS . Microbiota alterations in acute and chronic gastrointestinal inflammation of cats and dogs. World J Gastroenterol. 2014;20:16489‐16497. 25469017 10.3748/wjg.v20.i44.16489 PMC4248192

[29] Singh P , Teal TK , Marsh TL , et al. Intestinal microbial communities associated with acute enteric infections and disease recovery. Microbiome. 2015;3:45. 26395244 10.1186/s40168-015-0109-2 PMC4579588

[30] Minamoto Y , Otoni CC , Steelman SM , et al. Alteration of the fecal microbiota and serum metabolite profiles in dogs with idiopathic inflammatory bowel disease. Gut Microbes. 2015;6:33‐47. 25531678 10.1080/19490976.2014.997612 PMC4615558

[31] Guard BC , Barr JW , Reddivari L , et al. Characterization of microbial dysbiosis and metabolomic changes in dogs with acute diarrhea. PLoS One. 2015;10:e0127259. 26000959 10.1371/journal.pone.0127259 PMC4441376

[32] Schoster A , Mosing M , Jalali M , et al. Effects of transport, fasting and anaesthesia on the faecal microbiota of healthy adult horses. Equine Vet J. 2015;48:595‐602. 26122549 10.1111/evj.12479

[33] Fernandes KA , Kittelmann S , Rogers CW , et al. Faecal microbiota of forage‐fed horses in New Zealand and the population dynamics of microbial communities following dietary change. PLoS One. 2014;9:e112846. 25383707 10.1371/journal.pone.0112846 PMC4226576

[34] Blackmore TM , Dugdale A , Argo CM , et al. Strong stability and host specific bacterial community in faeces of ponies. PLoS One. 2013;8:e75079. 24040388 10.1371/journal.pone.0075079 PMC3770578

[35] Tedjo DI , Jonkers DM , Savelkoul PH , et al. The effect of sampling and storage on the fecal microbiota composition in healthy and diseased subjects. PLoS One. 2015;10:e0126685. 26024217 10.1371/journal.pone.0126685 PMC4449036

[36] Vandeputte D , Tito RY , Vanleeuwen R , Falony G , Raes J . Practical considerations for large‐scale gut microbiome studies. FEMS Microbiol Rev. 2017;41:S154‐S167. 28830090 10.1093/femsre/fux027 PMC7207147

[37] Weese JS , Jalali M . Evaluation of the impact of refrigeration on next generation sequencing‐based assessment of the canine and feline fecal microbiota. BMC Vet Res. 2014;10:230. 25267144 10.1186/s12917-014-0230-7 PMC4189626

