# Supplementary material for: Dysbiosis is not present in horses with fecal water syndrome when compared to controls in spring and autumn
Source: J Vet Intern Med. 2020 Jun 26;34(4):1614–21. doi: 10.1111/jvim.15778 (PMC7379055; doi:10.1111/jvim.15778)
Supplement: Supplementary file 1 — Appendix S1. Supplemental Table 1. Information to dysbiosis not present in horses with fecal water syndrome when compared to controls in spring and autumn: Population data on horses studied [file JVIM-34-1614-s001.pdf]

Supplemental Table 1. Information to dysbiosis not present in horses with fecal water syndrome when compared to controls in spring and autumn: Population data on horses studied

Horse	Farm	Sex	Breed	Year of birth	T1	T2	Health status	EGUS Grade [20]	ACTH Stim [11]	McMaster coprology EpG	Duration FWS
1	1	M	WB	2009	x	x	FWS	1	Normal	50	4 years
2	1	M	WB	2007	x	x	Control		Normal	0	
3	2	M	Freiberger	1996	x	x	FWS	0	Normal	0	> 1 year
4	2	M	Freiberger	1999	x		Control	0		0	
5	2	M	Freiberger	2007	x		Control		Normal		
6	3	G	Haflinger	1996	x		FWS			1250	
7	3	M	Haflinger	1998	x		Control		Normal	1300	
8	4	M	Freiberger	1999	x		FWS	0	Normal	450	Unknown
9	4	G	PRE	2005	x		Control				
10	5	G	WB	2001	x		FWS		Normal		
11	5	M	WB	2003	x		Control		Normal		
12	6	M	Freiberger	1994	x	x	FWS	0	Normal	0	> 1 year
13	6	M	Freiberger	1999	x	x	Control	0	Normal	0	
14	7	M	Arab-Frisian	2012	x	x	FWS	0	Normal		
15	7	M	Frisian	1994	x	x	Control				
16	8	M	Freiberger	1999	**x**		FWS	1	Normal		Since foal
17	8	M	WB	1997	x	x	Control				
18	9	G	WB	2007	x	x	FWS	1	Normal		Since foal
19	9	G	WB	2011	x		Control		Normal		
20	10	G	WB	2001	x	x	FWS	1			
21	10	M	WB	2006	x	x	Control		Normal		
22	11	G	Donkey	2002	x		FWS	1		0	3 years
23	11	G	Freiberger	2008	x	x	FWS	0	Normal	0	3 years
24	11	M	WB	2000	x	x	Control			0	
25	12	G	Icelandic	1982	x		FWS			0	1 year
26	12	M	Icelandic	2007	x	x	FWS	3	Normal	0	
27	12	G	Icelandic	1998	x	x	Control	1			
28	13	M	Freiberger	2005	x		FWS	0		50	9 years
29	13	M	Freiberger	2007	x		Control	0		0	
30	14	M	Fjord	2006	x		FWS	0		0	1 year
31	14	M	Fjord	2006	x		Control				

WB: Warmblood, TB: Thoroughbred, M: Mare, G: Gelding,
T1: Spring sample, T2: autumn sample
EGUS: Equine gastric ulcer disease, ACTH Stim: ACTH Stimulation and cortisol measurement in saliva
