# Supplementary figures and images for: Dysbiosis is not present in horses with fecal water syndrome when compared to controls in spring and autumn
Source: J Vet Intern Med. 2020 Jun 26;34(4):1614–21. doi: 10.1111/jvim.15778 (PMC7379055; doi:10.1111/jvim.15778)

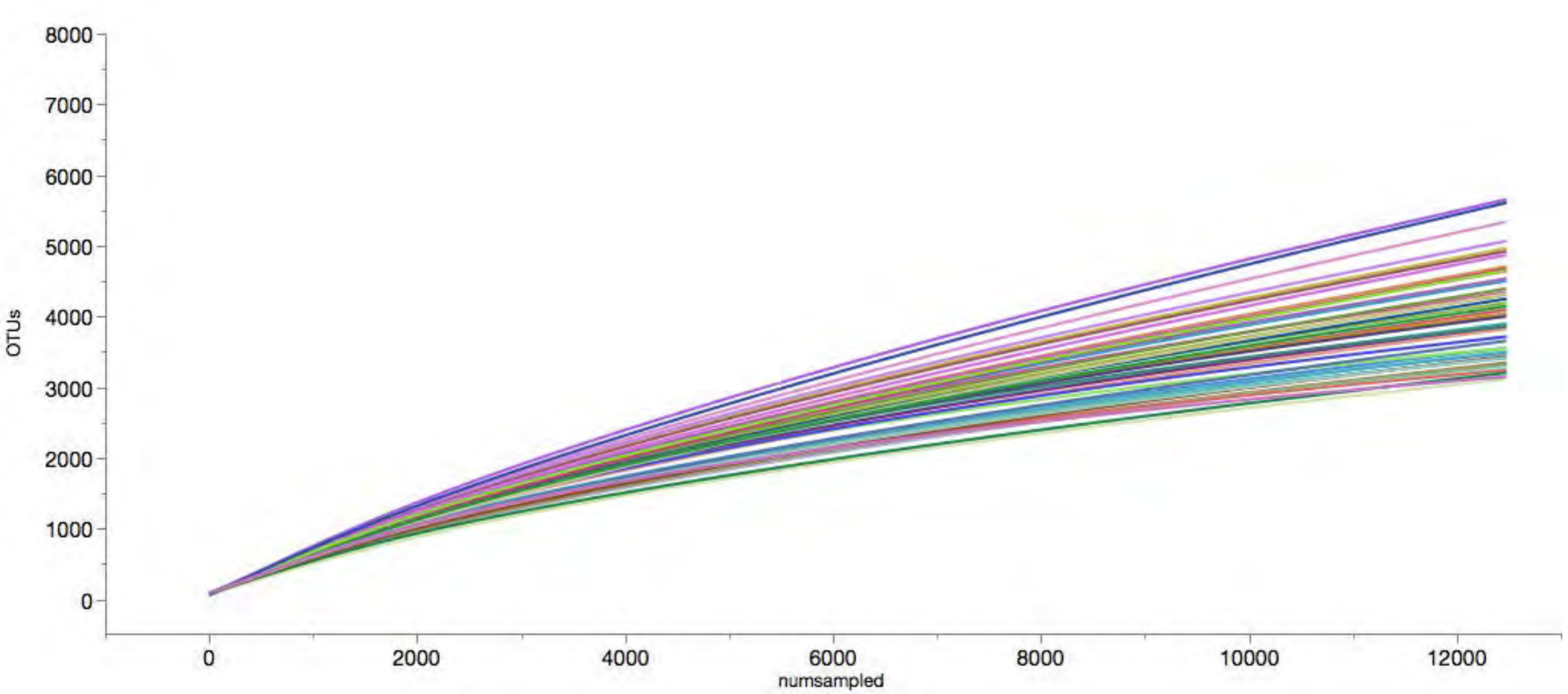

Supplement: Supplementary file 2 — Appendix S2. Supporting Information. [file JVIM-34-1614-s002.pdf]
