# Supplementary material for: Dysbiosis is not present in horses with fecal water syndrome when compared to controls in spring and autumn
Source: J Vet Intern Med. 2020 Jun 26;34(4):1614–21. doi: 10.1111/jvim.15778 (PMC7379055; doi:10.1111/jvim.15778)
Supplement: Supplementary file 3 — Appendix S3. Supplementary Table 2. LEFSE results [file JVIM-34-1614-s003.pdf]

Supplementary Table 2. LEFSE results

Season	Health	LDA	p-Value	OTU Name	Ruminococaceae or Lachnospiraceae
Spring	Control	3.44795	0.0158562	Unclassified bacteria	No
Spring	Control	3.08783	0.037867	Unclassified bacteria	No
Spring	FW	3.16298	0.0320798	Selenomonas	No
Spring	Control	2.90032	0.0281364	Unclassified Subdivison 5 Verrucomicrobia	No
Spring	FW	2.76736	0.0325703	Clostridium Ixa	Yes
Spring	FW	2.88301	0.0389951	Unclassified Lachnopsiraceae	Yes
Spring	FW	2.52464	0.02147	Unclassified Clostridia	No
Spring	Control	2.29873	0.0374802	Unclassified Subdivison 5 Verrucomicrobia	No
Spring	FW	2.43895	0.0433888	Oscillibacter	Yes
Spring	Control	2.36537	0.00924474	Unclassified bacteria	No
Spring	FW	2.10645	0.0313114	Unclassified Clostridiales	No
Spring	FW	2.08108	0.0182637	Unclassified Subdivison 5 Verrucomicrobia	No
Spring	FW	2.16426	0.0064183	Unclassified Clostridiales	No
Autumn	Control	2.53962	0.0174701	Stenotrophomonas	No
Autumn	Control	3.05904	0.0429504	Unclassified Firmicute	No
Autumn	Control	2.613	0.0362292	Mogibacterium	No
Autumn	Control	2.28283	0.0171686	Lactococcus	No
Autumn	FW	2.32737	0.0103358	Unclassified Ruminococcus	Yes
Autumn	Control	2.51352	0.0411809	Unclassified Ruminococcus	Yes
Autumn	Control	2.65492	0.0402639	Unclassified Gammaproteobacteria	No
Autumn	Control	2.22108	0.0413648	Brevundimonas	No
Autumn	FW	2.13933	0.030639	Aestuariispira	No
Autumn	Control	2.34219	0.00557011	Unclassified Ruminococcus	Yes
Autumn	Control	2.14656	0.033565	Unclassified Clostridiales	No
Autumn	Control	2.22307	0.044531	Unclassified bacteria	No
Autumn	Control	2.14869	0.0254365	Unclassified Erysopelotrichaceae	No
Autumn	Control	2.49805	0.0168274	Treponema	No
Autumn	FW	2.17382	0.038099	Unclassified Subdivison 5 Verrucomicrobia	No
Autumn	FW	2.11084	0.0367139	Unclassified bacteria	No

Autumn	FW	2.07548	0.0402162	Unclassified bacteria	No
Autumn	FW	2.12913	0.0499254	Unclassified Ruminococcus	Yes
Autumn	FW	2.08544	0.0377172	Unclassified Firmicute	No
Autumn	Control	2.16533	0.00560645	Treponemua	No
