# Supplementary material for: Dysbiosis is not present in horses with fecal water syndrome when compared to controls in spring and autumn
Source: J Vet Intern Med. 2020 Jun 26;34(4):1614–21. doi: 10.1111/jvim.15778 (PMC7379055; doi:10.1111/jvim.15778)
Supplement: Supplementary file 4 — Appendix S4. Supporting Information. [file JVIM-34-1614-s004.pdf]

☐ Les données du cheval doivent rester anonymes (pas de nom, ni de N° UELN)

☐ Les données du propriétaire doivent rester anonymes.

Questionnaire « selles liquides » 2013

Signalement

- Numéro UELN: ______________________________
- Nom du cheval: ______________________________
- Signalement (Race, Sexe, Age, Couleur): ______________________________
  ____
- Nom du propriétaire: ______________________________
- Lieu de détention (canton): ☐ AG ☐ BE ☐ BL ☐ BS ☐ FR ☐ JU ☐ LU ☐ NE ☐ SO ☐ VD ☐ VS ☐ autre:__________________
- Origines/Pedigree: Père: __________________; Mère: ________________; Père du père: _______________, Père de la mère: ______________
- Nombre de poulain: ______________________________
- Depuis quand le cheval vous appartient-il ou depuis quand le connaissez-vous? ______________________________
- Qui s'occupe de votre cheval? ☐ vous-même ☐ en pension
- Combien d'heures par jour en moyenne, votre cheval est-il sous la surveillance d'une personne?
- ☐ <4h ☐ 4-8h ☐ 8-12h ☐ >12h
- Caractère du cheval: ☐ calme, serein ☐ dynamique mais coopérant ☐ nerveux, pas très coopérant

Questions concernant les selles liquides
1ère partie: caractérisation

Depuis quand le problème des selles liquides existe-t-il chez votre cheval ?______________________________

Quelle structure ont les crottins de votre cheval? ☐ normale ☐ mouillée ☐ Diarrhée ☐ variable

Comment caractériseriez-vous les crottins de votre cheval sur une échelle de 1 (= odeur normale) à 5 (= malodorant)?

☐ 1 ☐ 2 ☐ 3 ☐ 4 ☐ 5

À quelle fréquence les selles liquides sont-elles observées?
☐ les selles liquides coulent en permanence (traces liquides en région anale)
☐ à chaque défécation, parfois les selles liquides sortent aussi entre deux défécations
☐ à chaque défécation
☐ intervalles irréguliers, mais plusieurs fois par jour
☐ intervalles irréguliers, mais un jour sur deux
☐ autre: ______________________________

Combien de temps une phase des selles liquides dure-t-elle? ☐ < 1 semaine ☐ 1-2 semaines ☐ 2-3 semaines ☐ >1 mois
☐ en permanence

Dans l'écurie, il y a-t-il encore d'autres chevaux souffrant du même problème? ☐ Oui ☐ Non

2ème partie: Association et causes possibles

Quand les selles liquides apparaissent-elles? ☐ au début de la saison de mise au pré ☐ à la fin de la saison de mise au pré ☐ quand il pleut
☐ en hiver ☐ en été ☐ aucune régularité décelable
☐ dans certaines situations* ☐ en permanence
* précisez: ☐ stress ☐ changement d'alimentation ☐ entraînement intensif ☐ transport

Si le stress est la cause: dans quelle situation votre cheval est-il stressé? ______________________________

____

Si le changement d'alimentation est la cause: quels fourrages ont un effet sur les selles liquides de votre cheval?
☐ herbe fraîche ☐ foin vieux et fibreux ☐ ensilage ☐ haylage ☐ aliment concentré
☐ pommes, carottes, etc. ☐ autre: ______________________________

3ème partie : Complications possibles

Votre cheval, présente-t-il des symptômes d'inconfort après avoir été à selle, lorsque les selles sont liquides (par ex. la queue reste en l'air longtemps après la défécation, rentre le ventre etc.)? ☐ Oui ☐ Non ☐ Ne sait pas

Est-ce que le cheval montre des symptômes des coliques lorsque les selles sont liquides ? ☐ Non, jamais ☐ rarement ☐ Oui, régulièrement

Le poids de votre cheval évolue-t-il? ☐ le cheval a maigri ☐ le cheval a pris du poids
☐ le poids est stable

Votre cheval souffre-t-il de lésions de la peau suite aux selles liquides? ☐ Oui ☐ Non
Caractérisez les lésions sur une échelle de 1 (pas de lésions) à 5 (lésions infectées) ☐ 1 ☐ 2 ☐ 3 ☐ 4 ☐ 5

Effectuez-vous un traitement, si oui lequel? ______________________________

____

Est-ce que les selles liquides de votre cheval vous dérangent (nettoyage, odeurs, complications etc.)? Caractérisez le dérangement sur une échelle de 1 (pas dérangeant) à 5 (vraiment dérangeant) ☐ 1 ☐ 2 ☐ 3 ☐ 4 ☐ 5

4ème partie: Diagnostique et thérapie

Avez-vous déjà contacté votre vétérinaire privé concernant ce problème des selles liquides? ☐ Oui ☐ Non

Quels examens ont été effectués?

☐ Coprologie
☐ Examen complet des crottins (analyse parasitologique, bactériologique, présence du sang dans les selles)
☐ Examen général du sang
☐ Biopsie rectale
☐ Gastroscopie
☐ Analyse du fourrage (valeurs alimentaires, teneurs en lignine et pectine etc.)
☐ Autre: ____

Quelle thérapie avez-vous déjà essayée afin de stoper les selles liquides de votre cheval?

	Résultat: Succès(+) ou échec (–)
☐ Changement de fourrage	______________________
☐ Donner de la luzerne	______________________
☐ Yoghourt au bifidus/ yoghourt nature	______________________
☐ Levure de bière	______________________
☐ Graines de lin, psilium	______________________
☐ Mash	______________________
☐ Produit de fermentation (z.b. Bokashi)	______________________
☐ Acupuncture	______________________
☐ Homéopathie	______________________
☐ Biorésonance	______________________
☐ Autre: ______________________	______________________

Maladies précédentes/actuelles

(L) Boiteries? ☐ Oui ☐ Non

→ Cause? ____________________

(R) Problèmes de dos? ☐ Oui ☐ Non

(H) Problèmes de nuque? (cervicaux) unilatéral ? raideur ? ☐ Oui ☐ Non

(K) Système gastro-intestinal? Coliques ? ☐ Oui ☐ Non

→ Cause? ☐ Nourriture ☐ à l'ârret ☐ autre

→ Opération? ☐ Oui ☐ Non

→ Si oui, combien de fois? ☐ 1 fois ☐ 2 fois ☐ 3 fois ☐ plus que 3 fois

(A) maladies respiratoires? ☐ Oui, RAO ☐ Oui, autre ☐ Non

(S) Eczéma d'été? ☐ Oui ☐ Non

(N) Urticaire? ☐ Oui ☐ Non

→ chronique, récidivant? ☐ Oui ☐ Non

(W) Verrues? (sarcoïdes?) ☐ Oui ☐ Non

(St) Stéréotypies?

→ Tic à l'ours? ☐ pas du tout ☐ de temps en temps ☐ régulièrement ☐ permanent, excessivement

→ Tic à l'appui? ☐ pas du tout ☐ de temps en temps ☐ régulièrement ☐ permanent, excessivement

→ Tic à l'air? ☐ pas du tout ☐ de temps en temps ☐ régulièrement ☐ permanent, excessivement

→ Tourner en rond? ☐ pas du tout ☐ de temps en temps ☐ régulièrement ☐ permanent, excessivement

→ Râper les dents contre le bois? ☐ pas du tout ☐ de temps en temps ☐ régulièrement ☐ permanent, excessivement

→ Autre? ____________________

(V) Problèmes comportementaux?

→ coller à d'autres chevaux? ☐ pas du tout ☐ de temps en temps ☐ régulièrement ☐ permanent, excessivement

→ mordre? ☐ pas du tout ☐ de temps en temps ☐ régulièrement ☐ permanent, excessivement

→ taper? ☐ pas du tout ☐ de temps en temps ☐ régulièrement ☐ permanent, excessivement

→ ruer? ☐ pas du tout ☐ de temps en temps ☐ régulièrement ☐ permanent, excessivement

→ pointer? ☐ pas du tout ☐ de temps en temps ☐ régulièrement ☐ permanent, excessivement

→ taper contre la paroi? ☐ pas du tout ☐ de temps en temps ☐ régulièrement ☐ permanent, excessivement

→ Autres: ____________________

→ Quelle situation provoque ce problème, en connaissez-vous l'origine ? ____________________

→ Avez-vous traité le problème? ____________________

Autres maladies? ☐ Non ☐ Wobbler ☐ Cushing ☐ Cœur/ système circulatoire ☐ Coup de sang

☐ Autre:____________________

Détention du cheval

Comment détenez-vous votre cheval? ☐ box intérieur ☐ box extérieur ☐ stabulation libre ☐ box avec sortie ☐ pré/abri

A-t-il des contacts avec des autres chevaux/avec des ânes ? ☐ avec des chevaux et ânes ☐ avec des chevaux ☐ avec des ànes
☐ non

Comment est-ce que vous sortez votre cheval? ☐ pré ☐ promenade ☐ paddock ☐ stabulation libre ☐ carrousel
→ À quel moment? ☐ le jour ☐ la nuit ☐ à l'aube
Depuis combien de temps le cheval est-il détenu de cette façon ?____________________________

Votre cheval vit-il dans un groupe des chevaux? ☐ Oui ☐ Non ☐ seulement au paddock ☐ seulement au pré
Où se situe votre cheval dans l'ordre hiérarchique du groupe ? ☐ animal de tête ☐ plutôt dominant ☐ plutôt soumis ☐ ne sait pas
Combien de fois la structure du groupe a-t-elle été modifiée? ☐ pas dans l'année précédente ☐ pas durant le semestre précédent
☐ durant le mois dernier ☐ chaque semaine

Combien d'heures par jour le cheval est-il dehors (pré, monté, attelé)? ☐ 0-1h ☐ 1-4h
☐ 4-12h ☐ > 12h

Quelle litière utilisez-vous? ☐ paille ☐ copeaux ☐ sciure ☐ chanvre ☐ tourbe ☐ pellets de paille
☐ autre: ____________________________

Utilisation

Comment utilisez-vous votre cheval? ☐ Loisirs ☐ Dressage ☐ Saut ☐ Military ☐ Western
☐ Attelage ☐ Endurance ☐ Courses ☐ Elevage ☐ Retraite
☐ Ecole ☐ autres: ____________________________

Combien de fois est-il bougé par semaine? ☐ < 1 fois ☐ 1-3 fois ☐ 4-5 fois ☐ 6 fois ☐ 7 fois

Est-ce qu'il va en concours? ☐ Oui ☐ Non
→ Combien de fois par année? ☐ 1-3 fois ☐ 4-8 fois ☐ 8-15 fois ☐ plus de 15 fois

À quelle fréquence montez-vous dans le manège? ☐ jamais/de temps en temps ☐ régulièrement (quotidiennement mais pas uniquement)
☐ toujours ☐ dépendant de la saison

Alimentation

Comment votre cheval est abreuvé? ☐ abreuvoir automatique ☐ seau ☐ fontaine
Où ce trouve l'abreuvoir automatique? ☐ juste à côté du place où le cheval mange ☐ dans l'autre coin du box/de la stabulation libre
L'abreuvoir, est-il chauffable? ☐ Oui ☐ Non ☐ s'il est gelé, on utilise des seaux

Fourrage? ☐ foin ☐ foin mouillé ☐ brique de foin ☐ paille ☐ silage ☐ haylage ☐ herbe
☐ autre: ______________________________
Fréquence? ☐ 1x ☐ 2x ☐ 3x ☐ plus

Aliment concentré? ☐ avoine ☐ mixte ☐ maïs ☐ orge ☐ autres: ______________________
Fréquence? ☐ 1x ☐ 2x ☐ 3x ☐ plus
Quantité (en kilo par jour)? ☐ <500g ☐ 500g – 1kg ☐ 1-3 kg ☐ 3-5 kg ☐ > 5 kg
Adaptez-vous la quantité d'aliment concentré selon le travail? ☐ Oui ☐ Non

Ordre de distribution de la nourriture? ☐ foin en premier ☐ aliment concentré en premier
Votre cheval est-il nourri différemment en été qu'en hiver (seulement concernant le fourrage)? ☐ Oui ☐ Non

Votre cheval, mange-t-il son foin dans le groupe? ☐ Oui ☐ Non
Est-ce que votre cheval se laisse chasser de la nourriture par les autres chevaux dans le groupe? ☐ Oui ☐ Non ☐ ne sait pas
Votre cheval, se bat-il pour la nourriture? ☐ Oui ☐ Non

Comment est l'appétit de votre cheval ? ☐ très bien, il mange tout ☐ modéré, parfois il mange avec des petites pauses
☐ insuffisant, le cheval mange seulement lentement et pas tout !

Est-ce que vous donnez des compléments nutritionnels ou médicamenteux? ☐ Non ☐ Vitamines, pierre à sel, minéraux
☐ pommes, carottes ☐ graines de lin, huile ☐ autres (préparations musculaires, supports pour les articulations etc.)
☐ médicaments
Pour quelle(s) raison(s) donnez-vous des médicaments ? ____

Faites-vous contrôler les dents de votre cheval ? ☐ Non ☐ Oui, 1-2x/année ☐ irrégulièrement (1x râpées/ plus depuis longtemps)
→ Par qui? ☐ Vétérinaire ☐ Dentiste équin ☐ Autre: ____________________

Faites-vous appel à d'autres mesures prophylactiques pour la santé de votre cheval? ☐ aucune ☐ Ostéopathie ☐ Acupuncture
☐ Homéopathie ☐ Physiothérapie ☐ autre: _________

Prévention

Contre quoi votre cheval est-il vacciné et combien de fois par année ?

Influenza (grippe équine)	□ jamais	□ 1fois	□ 2fois	□ 3fois ou plus
Tétanos	□ jamais	□ 1fois	□ 2fois	□ 3fois ou plus
EHV (Herpès)	□ jamais	□ 1fois	□ 2fois	□ 3fois ou plus
Rage	□ jamais	□ 1fois	□ 2fois	□ 3fois ou plus
Botulisme	□ jamais	□ 1fois	□ 2fois	□ 3fois ou plus
Autre: ____________________________	□ jamais	□ 1fois	□ 2fois	□ 3fois ou plus
Ne sait pas	□			

À quelle fréquence vermifugez-vous votre cheval par année? □ jamais/ irrégulièrement □ 1fois □ 2fois □ 3fois ou plus
□ hiver □ printemps □ été □ automne

→ Avec quoi vermifugez-vous votre cheval? □ ne sait pas □ en alternance □ toujours le même

- □ Benzimidazole (par ex. Panacur, Telmin, Equitac, Equimax, Vermitan, Rintal)
- □ Pyrimidine (Pyrantel) (par ex. Strongid)
- □ Pipérazine (par ex. Escovermin, Nemasin, Piperazin-citrat)
- □ Ivermectine/ Moxidectine (par ex. Equest, Eraquell, Noromectin, Equimax, Equalan)
- □ Praziquantel (par ex. Equimax, Equalan Duo)
- □ Autre: ____

Avez-vous déjà fait faire une coprologie (recherche de parasites dans les crottins)? □ Oui □ Non
A quelle fréquence? ____
Avec quel résultat? ____
Appliquez-vous d'autres stratégies pour combattre les vers? □ Non □ Ail □ Levure □ autre: _________________________

Gestion du pré? □ rien □ enlever les crottins □ grand pré □ pré individuel pour chaque cheval
□ changement de pré (seulement entre chevaux) □ changement de pré avec d'autres espèces □ pas de sortie au pré
→ Le pré est-il utilisé par d'autres espèces? □ Oui □ Non
→ Simultanément ou l'une après l'autre? □ simultanément □ l'une après l'autre
→ Quelles espèces? □ bovins □ moutons □ lamas/alpagas □ chèvres □ ânes □ mules □ cochons
□ autre: ____________________________________
→ Le pré est-il aussi fauché? □ Oui □ Non □ Ne sait pas
→ À quelle fréquence? □ jamais □ 1 fois □ 2 fois □ 3 fois ou plus
